# Multi-omics to predict changes during cold pressor test

**DOI:** 10.1186/s12864-022-08981-z

**Published:** 2022-11-19

**Authors:** Lisette J. A. Kogelman, Madeleine Ernst, Katrine Falkenberg, Gianluca Mazzoni, Julie Courraud, Li Peng Lundgren, Susan Svane Laursen, Arieh Cohen, Jes Olesen, Thomas Folkmann Hansen

**Affiliations:** 1 Danish Headache Center, Department of Neurology, Copenhagen University Hospital, 2600 Glostrup, Denmark; 2 Section for Clinical Mass Spectrometry, Danish Center for Neonatal Screening, Department of Congenital Disorders, Statens Serum Institut, Copenhagen, Denmark; 3 Novo Nordisk Foundation Center for Protein Research, Faculty of Health and Medical Sciences, University of Copenhagen, Copenhagen, Denmark

**Keywords:** Cold pressor test, Transcriptomics, Metabolomics, PCA, PLS, Systems biology, Data integration, Multi-omics

## Abstract

**Background:**

The cold pressor test (CPT) is a widely used pain provocation test to investigate both pain tolerance and cardiovascular responses. We hypothesize, that performing multi-omic analyses during CPT gives the opportunity to home in on molecular mechanisms involved. Twenty-two females were phenotypically assessed before and after a CPT, and blood samples were taken. RNA-Sequencing, steroid profiling and untargeted metabolomics were performed. Each ‘omic level was analyzed separately at both single-feature and systems-level (principal component [PCA] and partial least squares [PLS] regression analysis) and all ‘omic levels were combined using an integrative multi-omics approach, all using the paired-sample design.

**Results:**

We showed that PCA was not able to discriminate time points, while PLS did significantly distinguish time points using metabolomics and/or transcriptomic data, but not using conventional physiological measures. Transcriptomic and metabolomic data revealed at feature-, systems- and integrative- level biologically relevant processes involved during CPT, e.g. lipid metabolism and stress response.

**Conclusion:**

Multi-omics strategies have a great potential in pain research, both at feature- and systems- level. Therefore, they should be exploited in intervention studies, such as pain provocation tests, to gain knowledge on the biological mechanisms involved in complex traits.

**Supplementary Information:**

The online version contains supplementary material available at 10.1186/s12864-022-08981-z.

## Background

The cold pressor test (CPT) is probably the most widely used pain provocation test in pain research [1]. It involves keeping one hand in ice water as long as possible or until 10 min have passed. The duration that the subject can tolerate is a quantitative measure of the individual’s pain tolerability, and physiological responses can measure the fitness of the cardiovascular system. What regulates pain tolerance and cardiovascular responses is poorly understood, but peripheral and central factors may be both at play.

Understanding the biochemical mechanisms of pain tolerance is unfortunately limited by the fact that blood is the only easily accessible tissue for experimental studies in humans. There are, however, several studies indicating that blood composition reflects not only peripheral factors but also changes in the central nervous system [2]. Transcriptomics reveal gene activation patterns, while metabolomics give a snapshot of the biochemical status. Together, they enable investigation of the molecular mechanisms at play [3] and with metabolomics being close to the actual phenotype, it has shown to have great predictive abilities [4]. There has been recent progress in the extremely complicated biostatistical analysis of multi-omics and in the analysis of repeated measurements which is the most effective way to elucidate transient changes such as those induced by CPT.

In the present study we take advantage of these new methodological developments in an analysis of multi-omics, including transcriptomics and metabolomics, of CPT. The study was part of a larger project in migraine patients and therefore the experimental subjects are migraine patients. There is no evidence, however, that migraine patients respond differently to CPT or have a different pain threshold than healthy individuals [5, 6]. We, therefore, believe that our results are representative of CPT in the population at large. This is the first multi-omics study of CPT and one of the first studies combining multi-omics with longitudinal measurements.

## Materials and methods

### Study population and design

We performed a cold pressor test (CPT) on migraine patients recruited at the Danish Headache Center, as part of a larger project. The subjects were diagnosed with migraine (with or without aura) according to the International Classification of Headache disorders criteria, female, aged 18–70 year, weighing 45–95 kg and of Danish ethnicity. The CPT was performed in accordance with Hines and Brown [7] on a migraine- and headache-free day and with no record of subsequent migraine or headache the following 24 h. Subjects immersed their right hand as long as tolerated in ice water (max 10 min). Before, directly after, and 60 min after the CPT the heart rate and blood pressure were monitored (i.e. physiological measures). Before and 60 min after the test a blood sample was taken from the cubital vein, which was used for measuring steroid, metabolite- and gene expression. We successfully obtained full ‘omics data in 22 individuals.

### Steroid level measurement

The steroids 17-hydroxyprogesterone, testosterone, androstenedione and cortisol were measured as previously described [8]. To optimize normal distribution of the steroids, cortisol was square root transformed and androstenedione, progesterone and testosterone were log transformed.

### RNA-Sequencing

RNA-Sequencing and processing were performed as previously described by deCODE Genetics [8, 9]. As previously described, normalization was performed using DESeq2 [10], with normalization of the count matrix for library size and gene-length using the average gene length-matrix resulting from kallisto [11]. Genes that were not expressed in 90% of the samples were removed and only protein-coding genes were retained (*n* = 15,940). A regularized log transformation was performed using DESeq2.

### Metabolomics

#### Sample preparation

All samples, including blank and pooled quality control samples were submitted to untargeted liquid chromatography-tandem mass spectrometry (LC-MS/MS) metabolomics measurements at Statens Serum Institut, Copenhagen, Denmark, between July 3, 2019 and July 5, 2019. Plasma samples were thawed at room temperature and metabolites were extracted by transferring 40 µL of plasma onto 96-well plates with addition of 130 µL of icy cold extraction buffer 1. Plates were sealed and shaken for 15 min, at 750 rpm and 25 °C and kept at -20 °C overnight. In a second extraction step, plates were centrifuged for 10 min at 3220 G and 4 °C and 90 µL of supernatant from each well were transferred to a new glass-coated plate. 58 µL of icy cold extraction buffer 2 were added, plates were heat-sealed, shaken for 15 min at 750 rpm and 25 °C, and then kept at -20 °C for 30 min. The plates were then centrifuged for 10 min at 3220 G and 4 °C. Finally, 71 µL of supernatant were transferred to a hard-shell polypropylene plate and dried in an evaporator under nitrogen at 60 L/min at room temperature for 1 h, heat-sealed and kept at -20 °C until use. Extraction buffer 1 consisted of 150 µL NSK-A (vendor), 150 µL NSK-B (vendor), 150 µL labeled L-proline, 14.445 mL methanol (vendor) and 2.1 mL water, whereas extraction buffer 2 consisted of acetonitrile (vendor). Pooled quality control samples were prepared, by adding equal aliquots of all samples. Blank samples were prepared by injecting phosphate-buffered saline (PBS) in a K2EDTA 10-mL tube (BD vacutainer #367,525, final K2EDTA concentration 1.8 mg/mL) using a blood sample syringe. 4 mL of this matrix was then spiked with 40 µL of protease and phosphatase inhibitor mix (Thermo Fischer Scientific #78,446, no extra EDTA added) and extracted following the same protocol as experimental samples.

#### LC-MS/MS method

The LC-MS/MS platform (Thermo Scientific) consisted of a Q-Exactive Orbitrap mass spectrometer coupled to a Dionex Ultimate 3000 UPLC with a binary pump, hot pocket column heater, and CTC Combi PAL autosampler. Elution was performed on a C18 reversed phase column and corresponding pre-column (Acquity UPLC BEH 130 Å, 1.7 μm, 2.1 × 100 mm, Waters Corporation, Waltham, MA, USA). The mobile phase consisted of solvent A (99.8% H2O and 0.2% FA) and B (99.8% ACN and 0.2% FA). Frozen extracts were reconstituted in 51 µL reconstitution solvent (A:B 98:2), heat-sealed, shaken for 15 min at 750 rpm and 25 °C and centrifuged for 10 min at 3220 G and 4 °C before being injected (20 µL). Samples were maintained at 4 °C in the autosampler and the column temperature was maintained at 50 °C. Samples were injected in random order together with blank and pooled quality control samples at regular intervals. The gradient of the mobile phase was as follows: 0–1 min, A:B 98:2 at 0.3 mL/min; 1–6 min, gradient ramp to A:B 2:98; 6-6.83 min, A:B 2:98 step; 6.83–7.17 min, flow ramp up to 0.5 mL/min; 7.17-8 min, A:B: 2:98 step. In between each sample the chromatographic column was cleaned with solvent C (24.95% H2O, 24.95% MeOH, 24.95% ACN, 24.95% IPA, and 0.2% FA) with the following gradient: 8–11 min, flow and gradient ramp to A:B:C 0:2:98 0.3 mL/min; 11–13 min, gradient ramp to A:B:C 98:2:0; 13–14 min, flow ramp to 0.5 mL/min; 14–15 min, A:B:C 98:2:0 step; 15–21 min, A:B:C 98:2:0 step back at 0.3 mL/min. Tandem mass spectrometric data was acquired from 0.2 min to 8 min, in positive ionization mode, using a Heated Electrospray Ionization (HESI-II) Probe. Settings were spray voltage: 3.8 kV, capillary temperature: 350 °C, sheath gas pressure: 32 psi, auxiliary gas flow: 8 arb. unit, and S-lens radio frequency level: 50%. For full MS, the parameters were as follows: scan range from 70 to 1050 *m/z*, microscans: 1, resolution: 70,000, AGC target: 1E6, max IT: 120 ms, and spectrum data type: profile. For MS2, the parameters were as follows: scan range from 200 to 2000 *m/z*, microscans: 1, resolution: 17,500, AGC target: 1E5, max IT: 80 ms, top 8, isolation window: 1.0 *m/z*, isolation offset: 0 *m/z*, collision energy: stepped NCE (17.5, 35.0, 52.5 eV), spectrum data type: profile, minimum AGC target: 1.6E3, intensity threshold: 2E4, apex trigger: 2 to 4 s, charge exclusion: >2, peptide match: off, exclude isotopes: on, and dynamic exclusion: 12.0 s.

#### Data processing

ThermoFisher .raw files were converted to the .mzML format using Proteowizard’s MSConvert v3.0 (ProteoWizard Software Foundation, Palo Alto, CA, USA) [12], and preprocessed by using MZmine v2.40.1 [13, 14]. First, the raw data was cropped, with chromatogram retention time from 0 to 7.3 min retained. Mass lists were created with MS1 intensity above 1E4 and MS2 intensity above 0 retained. The chromatogram was built through the ADAP chromatogram builder [15], by using the following parameters: minimum group size of scans: 3, group intensity threshold 1E4, minimum highest intensity 3E4, and *m/z* tolerance 0.01 *m/z* or 10 ppm. The chromatogram was further deconvoluted by using the following parameters: *m/z* range for MS2 paring: 0.01 Da, RT range for MS2 paring: 0.4 min, and wavelets (ADAP) algorithm (S/N threshold: 10, S/N estimator: intensity window SN, minimum feature height: 3E4, coefficient/area threshold: 110, peak duration range: 0.05-1 and RT wavelet range 0.05–0.1). The peaks were deisotoped by using the isotopic peak grouper function, with the following parameters, *m/z* tolerance: 0.01 *m/z* or 10 ppm, retention time tolerance: 0.5 min, maximum charge: 3, representative isotope: most intense. Finally, the peaks from all samples were aligned by using the join align function with the following parameters: *m/z* tolerance: 0.01 *m/z* or 10 ppm, retention time tolerance: 0.5 min, weight for *m/z*: 75, weight for RT: 25. Features with apex between 0 and 0.4 min were filtered out. The generated MS/MS filtered feature table was exported in the .csv format and used for statistical analysis whereas aggregated MS/MS spectral information was exported in the .mgf format for chemical structural annotation through the feature-based mass spectral molecular networking workflow within the Global Natural Products Social Molecular Networking Platform (GNPS) [16, 17].

Before statistical analysis, relative intensities in the MS/MS filtered mass spectral feature table were scaled by dividing each mass spectral feature by its batch root mean square using R’s scale function to normalize for batch effect [18]. Filtering of mass spectral features per sample was based on a 20-fold difference between blank and experimental samples, and metabolite-level features were filtered based on the modified 80% rule (i.e. mass spectral features present in at least 80% of the samples per experimental group). This resulted in inclusion of 622 mass spectral features with associated MS/MS fragmentation spectrum, which we here refer to as a proxy for metabolites.

#### Chemical structural identification

A mass spectral molecular network was created through the Global Natural Products Social Molecular Networking Platform (GNPS) (http://gnps.ucsd.edu) using the feature based molecular networking workflow (https://ccms-ucsd.github.io/GNPSDocumentation/featurebasedmolecularnetworking/) [16]. The data was filtered by removing all MS/MS fragment ions within +/- 17 Da of the precursor *m/z*. MS/MS spectra were window filtered by choosing only the top 6 fragment ions in the +/- 50Da window throughout the spectrum. The precursor ion mass tolerance was set to 0.02 Da and a MS/MS fragment ion tolerance of 0.02 Da. A network was then created where edges were filtered to have a cosine score above 0.7 and more than 4 matched peaks. Further, edges between two nodes were kept in the network if and only if each of the nodes appeared in each other’s respective top 10 most similar nodes. Finally, the maximum size of a molecular family was set to 100, and the lowest scoring edges were removed from molecular families until the molecular family size was below this threshold. The spectra in the network were then searched against GNPS’ spectral libraries. The library spectra were filtered in the same manner as the input data. All matches kept between network spectra and library spectra were required to have a score above 0.7 and at least 4 matched peaks. Results were visualized using Cytoscape v3.5.0 [19]. To further enhance chemical structural information, MS2LDA substructure information (https://ccms-ucsd.github.io/GNPSDocumentation/ms2lda/) [20] and information from *in silico* structure annotation from Network Annotation Propagation [21] were incorporated within the GNPS mass spectral molecular network using the MolNetEnhancer workflow (https://ccms-ucsd.github.io/GNPSDocumentation/molnetenhancer/) [22]. In addition, MS/MS fragmentation spectra were searched using the *in silico* tools SIRIUS + CSI:FingerID [23, 24] and CANOPUS [25, 26] as well as the mass spectrometry search tool MASST [27]. Links to chemical structural information retrieved through the GNPS feature based, MS2LDA, Network Annotation Propagation, MolNetEnhancer and MASST workflows are available upon request.

### Principal component analysis

A principal component analysis (PCA) was performed on each data level (i.e. physiological measures, steroids, metabolomics, and transcriptomics) to investigate the major source of variation in each data set. With respect to the paired-sample design, we performed a multi-level PCA on the within-subject deviation matrix using the pca() function of the R-package mixOmics, by giving the subject’s ID to the multilevel setting [28, 29].

### Sparse partial least squares – discriminant analysis

Multilevel sPLS-DA is a supervised approach utilizing the paired-sample design whereby variation for the response variable is optimized. Further, this linear multivariate approach takes the dependencies between genes into account which reduces data dimensionality, which is especially useful for data sets with a large number of features on a relatively small number of samples. sPLS-DA was performed using the splsda() function of R-package mixOmics [28, 30]. In the case of transcriptomics and metabolomics, the number of features and components were optimized using leave-one-out cross-validation. Based on the classification error rate, the number of components were chosen, and tuning of the model was performed to minimize the number of features per component while maximizing the class discrimination. Based on the lowest error rate, the optimal number of features per component were selected for the final sPLS-DA model. The performance of the final model was evaluated using the perf() function with a leave-one-out validation, resulting in error rates of the components. The workflow was followed as presented in the tutorials of mixOmics (http://mixomics.org/case-studies/). Given the number of physiological and steroids measures, no selection was done on them. Physiological measures taken right after CPT were used, as they display the direct effect of CPT. A model was called significant when *P* < 0.05 and the area under the operator curve (AUC) and error rates were presented as evaluation indexes. We note that the AUC will be overestimated, as we did not have access to a validation cohort, and instead, a ‘leave-one-out’ performance test was performed.

### PLS-DA on pathway level

To include prior biological knowledge, we transformed the gene-level expression dataset into a KEGG pathway-level dataset, by extracting the module eigengene of each pathway using the WGCNA R-package [31]. The module eigengene represents the first principal component of a set of genes. The sPLS-DA on the pathway-based dataset allows identification of pathways affected between the two time points.

### Multilevel multi-omics analysis: DIABLO

Data Integration Analysis for Biomarker Discovery (DIABLO), is a method developed within mixOmics, integrating different ‘omics data levels using a PLS approach [32]. The method aims to identify correlated features explaining the outcome of interest (i.e. changes due to CPT). To increase the power of the paired-sample design, we first extracted the within-subject deviation matrices per ‘omics data level. Next, using the function block.splsda, parameters were tuned to select the optimal number of components and features per components while minimizing the error rate. A full design was used to identify correlations between the different ‘omics levels; a correlation threshold of 0.8 was used to identify biologically relevant features. The workflow was followed as presented in tutorials of mixOmics (http://mixomics.org/mixdiablo/).

### Differential expression analysis

Differential gene expression analysis was performed as a paired sample design. Differentially expressed (DE) genes between the two time points (before and after CPT) were detected using a binomial Wald Test within the R-package DESeq2 [10], with individual patient ID as covariate. P-values were adjusted for 15,918 tests using Bonferroni correction (P_adj_) and were called DE in case P_adj_ < 0.05.

Differential metabolite expression analysis was likewise performed as paired sample design. DE metabolites were detected using a Wilcoxon signed-rank test. P-values were adjusted for 622 tests using the false discovery rate (FDR) and were called DE in case FDR < 0.05.

### Gene set enrichment analysis

Enriched pathways of sets of genes (i.e. DE genes and clusters of genes found by sPLS-DA and DIABLO) were detected using the STRING database (v11.0) [33]. P-values were adjusted for multiple testing using FDR. Enriched tissues were detected using FUMA using the GENE2FUNC function, using default settings [34]. Pathways and tissues were called enriched when FDR < 0.05.

## Results

### Clinical descriptive statistics

The cold pressure test (CPT) was performed on 22 individuals diagnosed with migraine, with a mean age of 36.5 years (SD = 11.3 years) and a mean BMI of 23.7 (SD = 3.3). Participants kept their hand in the ice water for an average of 283 s (SD = 222 s) ranging from 21 s to the predefined maximum of 600 s. Compared to before the CPT, the systolic blood pressure was significantly increased as measured immediately after CPT (*P* = 0.02), while diastolic blood pressure and heart rate were not. No differences in blood pressure or heart rate were detected comparing before the CPT with 60 min after CPT.

### Principal component analysis

**Fig. 1:**
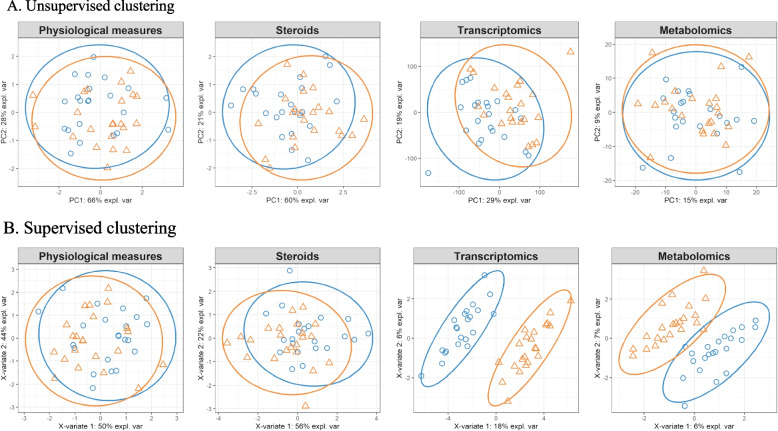
Clustering of the ‘omics samples before (blue circles) and after (orange triangles) the cold pressor test using **A**) multilevel PCA (unsupervised clustering) and **B**) multilevel sPLS-DA (supervised clustering). No significant prediction between the time points could be made using PCA, though using sPLS-DA both transcriptomics and metabolomics enabled significant prediction of before vs. after cold pressor test

Dimensionality reduction of the samples using a multilevel Principal Component Analysis (PCA) did not allow to distinguish the two time points at any ‘omics data level (see Fig. 1A). Thus, no overall mechanism of CPT could be found.

### Sparse partial least squares – discriminant analysis

Dimensionality reduction using sparse Partial Least Squares – Discriminant Analysis (sPLS-DA) clearly distinguished before and after CPT in transcriptomics and metabolomics, but not in physiological measures or steroids (Fig. 1B).

#### Physiological measures

Physiological measures before and right after CPT (heart rate, systolic and diastolic blood pressure) did not discriminate the time points, i.e. with one component the AUC was 0.59 (*P* = 0.30) with an error rate of 0.45. From single-variable analysis, the heart rate was the most predictive measure with an AUC of 0.57 (*P* = 0.40).

*Steroids.* The steroids progesterone, testosterone, androstenedione and cortisol were measured before and after CPT with optimal prediction using all four steroid levels (two components). The components were not able to discriminate the time points and had an AUC of 0.64 (*P* = 0.12).

#### Transcriptomics

The expression levels of > 15 K genes were reduced in dimension by tuning the number of components and features per components, leading to component one to four consisting of 20, 5, 30 and 5 genes. Those four components were able to significantly discriminate the time points with an AUC of 0.92 (*P* = 1.50 × 10^− 6^) with an error rate of 0.14, 0.09, 0.05, and 0.09 for the four components. Enrichment analysis with String-db showed enrichment of the Reactome pathways; *Metabolism of lipids* (FDR = 0.01), *WNT mediated activation of disheveled (DVL)* (FDR = 0.04), *Phospholipid metabolism* (FDR = 0.04), *Metabolism* (FDR = 0.04), and the UniProt keyword: *Phosphoprotein* (FDR = 0.04). Tissue enrichment analysis with FUMA showed *whole blood* and *heart left ventricle* as significantly expressed tissues (P_bonf_ < 0.05); no brain tissues were significantly expressed.

Next, we performed sPLS-DA on pathway level. After tuning, one component with one feature was selected as the best model to distinguish the two time points. The KEGG pathway *Pantothenate and coenzyme A (CoA) biosynthesis (map00770)* distinguished the two time points with an AUC of 0.81 (*P* = 3.59 × 10^− 4^), and an error rate of 0.14. This pathway included 6 genes: *DPYD, ENPP1, ENPP3, UPB1, PANK1* and *PANK4*. None of the genes were part of the components detected by the sPLS-DA on gene expression.

#### Metabolomics

The metabolomic dataset consisted of 622 metabolites. It was reduced in dimension by tuning of the parameters to component one and two, both consisting of five metabolites. This resulted in a significant discrimination of the time points with an AUC of 0.89 (*P* = 1.02 × 10^− 5^) and an error rate of 0.32 and 0.32 for the two components. Partial putative chemical structural annotation could be retrieved for one metabolite in component 1 and two metabolites in component 2 (Additional file 1), corresponding to a level 3 metabolite identification according to the Metabolomics Standard Initiative’s reporting standards [35]. One metabolite in component 1 was indicative of a disaccharide structure (ID 6108), whereas one metabolite in component 2 could be putatively annotated as catecholamine (ID 7059), based on its structural relatedness to phenylethanolamine. A third metabolite (ID 4001) was putatively annotated as peptide containing a phenylalanine substructure, based on its structural relatedness to multiple high molecular weight compounds and presence of a phenylalanine-COOH substructure.

### Integration of the different ‘omics layers

Physiological measures did not significantly discriminate the time points in clustering analysis. As they are conventionally used in the CPT, we tried to integrate them with metabolomics and transcriptomics data. Correlations estimated by PLS between the different ‘omics levels ranged from 0.39 (steroids vs. transcriptomics) to 0.87 (metabolomics vs. transcriptomics). After tuning parameters within DIABLO, two components were selected which both had 3, 4, 10, and 20 features (physiological measures, steroids, metabolomics and transcriptomics). Correlations between the ‘omics levels increased and ranged from 0.46 (physiological measures vs. steroids) to 0.88 (metabolomics vs. transcriptomics), resulting in an AUC of 0.96 (*P* = 2.61 × 10^− 5^) explained by the two components.

Focusing on integrated metabolomics and transcriptomics, which were able to significantly distinguish the time points, the tuning to select features did not change the correlation between metabolomics and transcriptomics (i.e. 0.87 both before and after tuning). We identified components one to three containing 10,10, and 10 metabolites (error rate of 0.32, 0.14, and 0.05) and 10,10, and 20 genes (error rate of 0.14, 0.05, and 0.00). Using those three components, the AUC was 0.99 (*P* = 2.68 × 10^− 8^).

The AUC of the model was higher and more significant when excluding physiological measures and steroid data levels, and therefore downstream analysis was performed on integration results of metabolomics and transcriptomics only. Enrichment analysis of the 40 genes showed enrichment of the Reactome pathway *Activation of PUMA and translocation to mitochondria* (FDR = 0.04) and the following tissues were significantly enriched: spleen, sigmoid colon, prostate, and coronary artery. Among the 30 metabolites, partial putative chemical structural annotation could be retrieved for 19 metabolites (Additional file 1). Five metabolites could be annotated through GNPS spectral library matching, manual annotation propagation throughout the network and SIRIUS + CSI:FingerID *in silico* structure annotation with an annotation level 2 [35], including two amino acids, methionine, arginine, an acylcarnitine, propionylcarnitine, two bile acids, glycochenodeoxycholate and taurohyodeoxycholic acid. Twelve metabolites could be putatively annotated at an annotation level 3. Six metabolites contained lipid and sugar substructures, suggestive of glycerophospholipids and glycerophosphoethanolamines, whereas two metabolites were suggestive of long-chain fatty acids and four metabolites of an alpha amino acid, a nucleobase, an acylcarnitine, and a peptide structural analogue respectively.

**Fig. 2:**
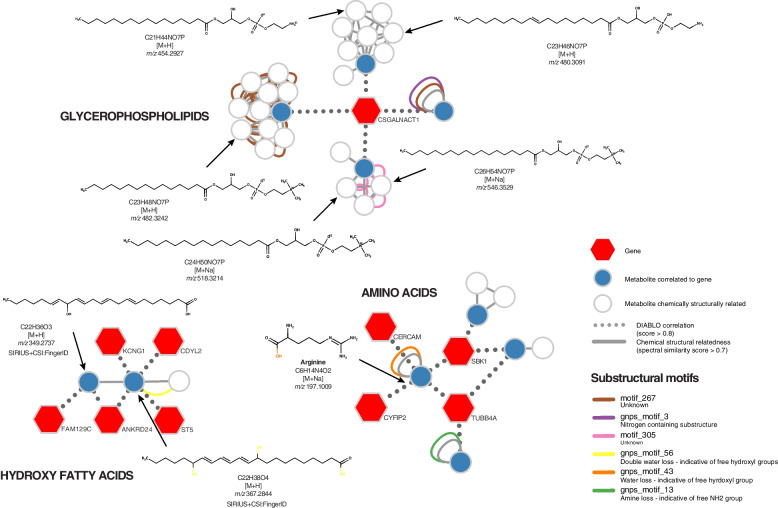
Network of correlating genes and metabolites, detected by DIABLO. Genes in blue, metabolite IDs in red

Several of the genes and metabolites are strongly correlated with each other (Fig. 2). Interestingly, we see that *CSGALNACT1* (Chondroitin Sulfate N-Acetylgalactosaminyltransferase 1) is correlated with four metabolites, of which three are suggestive of glycerophospholipid structural analogues and one is suggestive of a glycerophosphoetanolamine structural analogue. *CSGALNACT1* encodes an enzyme transferring N-acetylglucosamine, a sugar donor for glycophosphatidylinositol lipid synthesis [36]. Based on the positive correlation between *CSGALNACT1* and the metabolites, we therefore speculate that the involved metabolites are glycophosphatidylinositol anchored proteins, containing both glycophosphatidylinositol as well as a phosphoethanolamine substructures. Another cluster suggests long chain fatty acids (ID 6316 and 5396) to correlate with the genes *FAM129C, ANKRD24, ST5, CDYL2* and *KCNG1*. No direct association could be found between those genes and long chain fatty acids and using the STRING database no direct associations were found between those five genes. Lastly, we found a cluster of an alpha amino acid structural analogue (ID 2536), arginine (ID 157), an acylcarnitine structural analogue (ID 1257) and a nucleobase structural analogue (ID 3029) with the genes *TUBB4A, CYFIP2, CERCAM* and *SBK1*.

### Differential expression analyses

**Fig. 3:**
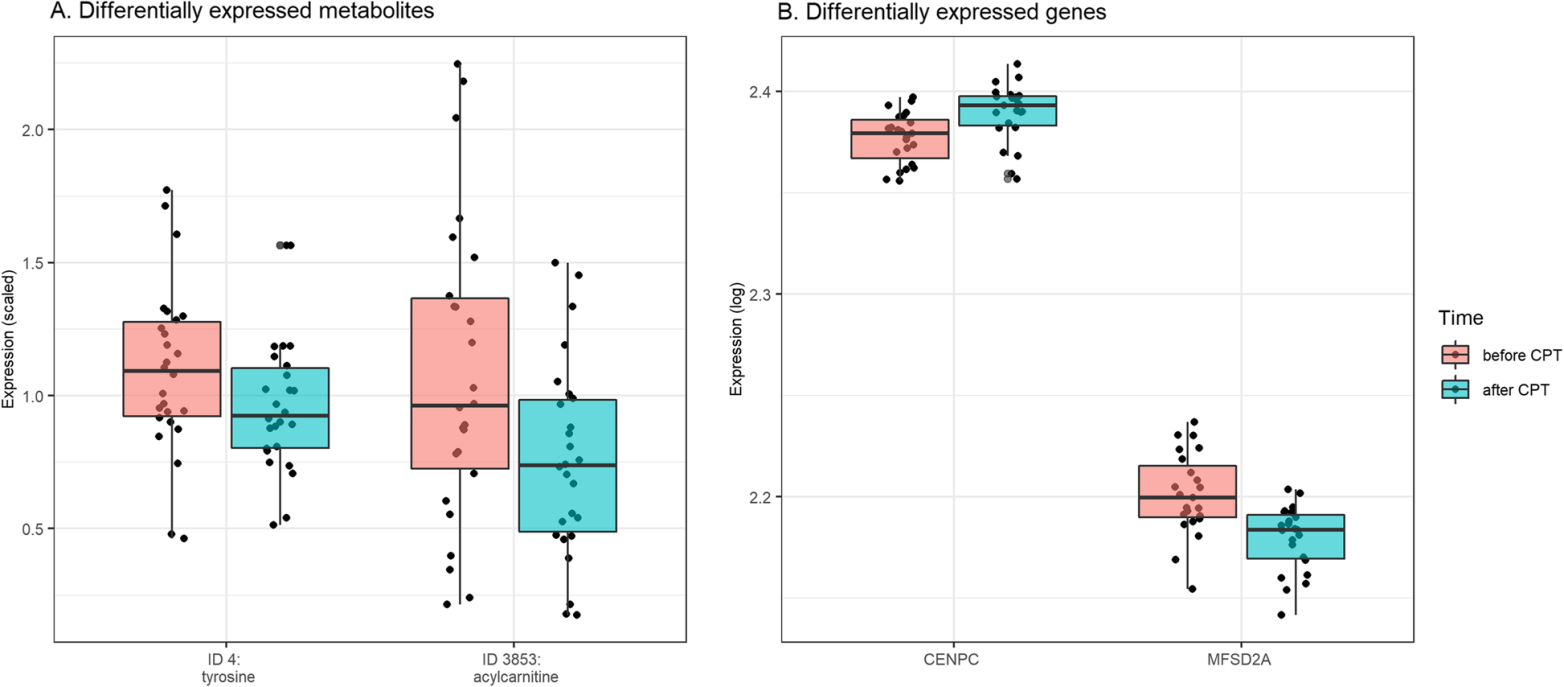
Two of the top differentially expressed metabolites (**A**) and genes (**B**) before vs. after cold pressor test (CPT)

Differential expression analysis of transcriptomics revealed 44 differentially expressed (DE) genes: ten were upregulated and 34 downregulated after CPT (Fig. 3B). Assessing the protein product of the DE genes, revealed an enrichment for the UniProt description for lipid transporting (FDR = 0.02) and anion exchange proteins (FDR = 0.03). Several of the DE genes correlated significantly with one or more metabolites (FDR < 0.05), of which putative chemical structural annotation at a level 2–3 could be retrieved for eight metabolites, two acylcarnitines, two amino acids, a carnitine, a fatty alcohol, a dicarboxylic acid and a glycosylated molecule: *CENPC* with oleoylcarnitine-C7H14 (ID 3853), *KLC2* and *NEMF* with creatine (ID 2043), *MFSD2A* with carnitine (ID 5), *PDK4* with O-Acetylcarnitine (ID 11), *RPGR* with a fatty alcohol structural analogue (ID 9097), a glycosylated molecule (ID 1279) and 5-amino-2-(cyclohexanecarbonylamino)-5-oxopentanoic acid (ID 6394), and *SMPD3* with a dicarboxylic acid structural analogue (ID 15,291).

Differential expression analysis of the metabolomics data revealed 21 DE metabolites, all were downregulated after CPT (Fig. 3 A). Among these 21 metabolites, partial putative chemical structural annotation could be retrieved for 14 metabolites at an annotation level 2–3 (Additional file 1). Eight metabolites could be annotated through GNPS spectral library matching, manual annotation propagation throughout the network and SIRIUS + CSI:FingerID *in silico* structure annotation with an annotation level 2 [35], including three amino acids (tyrosine, methionine, creatine), one indole (tryptophan) and one acylcarnitine (propionylcarnitine). Six metabolites were indicative of steroid, tryptophan, acylcarnitine, glycerophospholipid, bile acid and phenylalanine structural analogues respectively. Several of the annotated metabolites were significantly correlated with transcriptomics (FDR < 0.05), i.e. tyrosine (ID 4) was correlated with *MMAB* (*r* = 0.63), creatine (ID 2043) with *LIPE* (0.67), *ZBTB32* (0.66), *CPNE5* (0.65) and more, oleoylcarnitine-C7H14 (ID 3853) with *IGFBP2* (*r* = 0.63) and *CENPC* (*r* = -0.62, FDR = 0.03), a glycerophospholipid structural analogue (ID 5380) with *GADD45G* (0.66), *SCARF1* (0.66), *NDST1* (0.64), and lastly, a acylcarnitine structural analogue (ID 5479) with *KCNH2* (0.61).

## Discussion

In this study we show the potential of multi-omics data integration in pain research, using samples taken before and after a cold pressor test (CPT). By analyzing data at a systems-level instead of only at feature-level we showed that ‘omics levels discriminate the time points before and after CPT better than conventional physiological measures. Over and above, we point to clusters of features which differ between the two time points, indicating that integration of ‘omics levels has a great potential to identify relevant molecular mechanisms.

The CPT has been used in research to investigate both pain response and cardiovascular response. Though a significant raise in systolic blood pressure was present after CPT, clustering analysis with PCA or PLS was not able to distinguish the two time points. Furthermore, when integrating the physiological measures with other data levels, the physiological measures were not contributing to the model in DIABLO and made prediction of the time points less accurate. This indicates that changes, i.e. effect sizes, in physiological measures during the CPT are small and/or our sample size was too small to discriminate the time points due to interindividual variation using PCA or PLS. Moreover, the physiological measures do not provide information on involved molecular mechanisms.

Common and complex traits are caused by environmental and genetic factors, and incorporation of the different ‘omics layers provides a more comprehensive understanding of the trait [37]. Partial Least Squares (PLS) analysis, a multivariate dimensionality reduction approach, has been developed recently allowing ‘omics analyses on systems-level. Importantly, it can handle multicollinearity of the features, which often occurs with metabolomic data [38]. PLS incorporates the relationships between features, reduces data dimensionality and provides information on which features are driving the biological signal. It has been advanced to PLS with discriminant analysis (PLS-DA), enabling prediction of categorical values and to sparse PLS-DA (sPLS-DA) which enables the selection of discriminating values [30]. To simplify: where PCA reduces dimensionality by detecting components that preserves as much of the variance in the data, PLS-DA preserves as much of the covariance between the data and phenotype of interest [39]. In the present study, we show the potential of sPLS-DA: both in transcriptomics and metabolomics we were not able to discriminate the time points using PCA, while sPLS-DA significantly distinguished the time points and detected features explaining this differentiation.

A challenge of untargeted metabolomic analysis is the incomplete chemical structural annotation; on average only 2–5% of all mass spectra collected in a typical LC-MS/MS experiment can be matched to known molecules [40]. Using a combination of several recent mass spectral data mining tools, we were able to annotate chemical structures or classes for almost 50% of our spectra of interest, corresponding to an annotation level 2–3 according to the Metabolomics Standard Initiative [35] (Additional file 1). Furthermore, we showed that annotation of metabolites can be enhanced by integration with transcriptomics data. For example, further evidence for annotation of three unknown metabolites could be collected based on their correlation with *CSGALNACT1*: metabolomic mass spectral annotation suggested lipid and sugar substructures, but based on the integration with transcriptomics data, we could propose a structural hypothesis supporting glycophosphatidylinositol lipids.

The CPT affects many different molecular mechanisms, e.g. stress/pain reaction and cardiovascular responses. Several analyses showed the involvement of lipid transport/metabolism. One of the DE genes present in the lipid transport pathway, a pathway significantly overrepresented in several transcriptomic analyses in this project, is *MFSD2A*. It correlated with carnitine, a metabolite playing an important role in transporting fatty acids into and out of the mitochondrion [41]. Lipid metabolism has a key role in pain mechanisms [42, 43] and cardiovascular pathways [44, 45]. Closely related is the regulation of glucose by insulin. One of the DE genes, *PDK4*, significantly correlated with O-acetylcarnitine. The expression of *PDK4* is regulated by, among others, insulin [46]. Furthermore, PDK4 inhibits the pyruvate dehydrogenase complex, which increases the influx of acetyl-coA from beta-oxidation into the Krebs cycle (and in turn, slowing glycolysis). Acetyl-L-carnitine together with coenzyme A (CoA) converts into acetyl-CoA, which is involved in insulin sensitivity [47]. Among the DE metabolites we found carnitine and carnitine-related metabolites. One acylcarnitine correlated with *IGFBP2*, encoding an insulin-like growth factor binding protein. Thus, both metabolites and genes point to the regulation of insulin during CPT. Furthermore, catecholamines, also found among the DE metabolites, are known to play a key role in stress response [48].

In conclusion, we here show the potential of multi-omics in pain research on both feature- and systems-level. Regardless of the small sample size, a high predictive ability was found for both metabolomics, transcriptomics, and their interaction. However, a replication cohort is required for validation. We suggest that future research, investigating pain and/or vascular response, considers investigation of ‘omics levels. Despite the fact that it’s in its infancy and the present study is, therefore, of a pioneering type, multi-omics analyses are certain to further improve insight into biological mechanisms nearby future.

## Supplementary Information

**Additional file 1.** Annotation of identified metabolites.

## Data Availability

The dataset generated and analyzed during the current study is available in the European Genome-Phenome Archive (EGA) under EGA ID: EGAS00001006690. [https://ega-archive.org/studies/EGAS00001006690]
